# Global health systems partnerships: a mixed methods analysis of Mozambique’s HPV vaccine delivery network actors

**DOI:** 10.1186/s12889-020-08958-1

**Published:** 2020-06-05

**Authors:** Caroline Soi, Jessica Shearer, Baltazar Chilundo, Vasco Muchanga, Luisa Matsinhe, Sarah Gimbel, Kenneth Sherr

**Affiliations:** 1 Department of Global Health, University of Washington, Harris Hydraulics Laboratory, 1510 San Juan Road, Seattle, WA 98195 USA; 2 Health Alliance International, 1107 NE 45TH St #350, Seattle, WA 98105 USA; 3 PATH, 2201 Westlake Ave, Seattle, WA 98121 USA; 4 Universidade Eduardo Mondlane, Av. Salvador Allende no. 702, Maputo, Mozambique; 5 Health Alliance International, Rua Caetano Viegas no. 67, Maputo, Mozambique; 6 Department of Family and Child Nursing, University of Washington, Magnuson Health Sciences Building, 1959 NE Pacific St, Seattle, WA 98195 USA

**Keywords:** Partnership, HPV vaccine, SNA, Mozambique, Gavi, Global health, Demonstration project

## Abstract

**Background:**

Global health partnerships have expanded exponentially in the last two decades with Gavi, the Vaccine Alliance considered the model’s pioneer and leader because of its vaccination programs’ implementation mechanism. Gavi, relies on diverse domestic and international partners to carry out the programs in low- and middle-income countries under a partnership engagement framework (PEF). In this study, we utilized mixed methods to examine Mozambique’s Gavi driven partnership network which delivered human papillomavirus (HPV) vaccine during the demonstration phase.

**Methods:**

Qualitative tools gauged contextual factors, prerequisites, partner performance and practices while a social network analysis (SNA) survey measured the partnership structure and perceived added value in terms of effectiveness, efficiency and country ownership. Forty key informants who were interviewed included frontline Ministry of Health workers, Ministry of Education staff and supporting partner organization members, of whom 34 participated in the social network analysis survey.

**Results:**

Partnership structure SNA connectivity measurement scores of *reachability (*100%) and average *distance* (2.5), were high, revealing a network of very well-connected HPV vaccination implementation collaborators. Such high scores reflect a network structure favorable for rapid and widespread diffusion of information, features necessary for engaging and handling multiple implementation scales. High SNA effectiveness and efficiency measures for *structural holes* (85%) and low *redundancy* (30%) coupled with high mean perceived effectiveness (97.6%) and efficiency (79.5%) network outcome scores were observed. Additionally, the *tie strength* average score of 4.1 on a scale of 5 denoted high professional trust. These are all markers of a collaborative partnership environment in which disparate institutions and organizations leveraged each entity’s comparative advantage. Lower perceived outcome scores for country ownership (24%) were found, with participants citing the prominent role of several out-of-country partner organizations as a major obstacle.

**Conclusions:**

While there is room for improvement on the country ownership aspects of the partnership, the expanded, diverse and inclusive collaboration of institutions and organizations that implemented the Mozambique HPV vaccine demonstration project was effective and efficient. We recommend that the country adapt a similar model during national scale up of HPV vaccination.

## Background

Health program implementation especially in low-and middle-income countries (LMICs), requires the involvement of different types of actors from different sectors [1]. Disparate individuals from different types of organizations and institutions, spanning multiple and hierarchical health system levels, such as community, health facility, regional, national and international all have a role to play [2, 3]. This diversity in actors relies on various relationships and interactions amongst all players involved in program operationalization. Partnership, which is defined as a collaboration with the mission of accomplishing a common goal either contractually or non-contractually, is a key type of such relationships [4]. Partnerships, have traditionally existed at the country level amongst different implementing entities, however the global health partnerships (GHPs) phenomenon, is more recent, with most such partnerships being created in just the last two decades [5]. Despite their novelty, GHPs have expanded exponentially and gained broader relevance to the extent that they are now a salient feature of health program implementation in LMICs [6, 7]. Consequently, partnership has become a significant health program implementation determinant in these settings [8] and the broader implementation science (IS) field formally recognizes the study and measurement of partnerships as a core component of implementation research [9–11]. The field’s practitioners argue that inter-organizational collaborations create larger pools of resources which lead to accelerated adoption, institutionalization and sustainment of global health programs [12].

Gavi, the Vaccine Alliance (Gavi), is a GHP that was set up with the agenda of increasing availability and accessibility of new and underutilized vaccines in LMICs [13]. Founded in 1999, the public private partnership brings together four founding organizations, the Bill and Melinda Gates Foundation (BMGF), World Bank, World Health Organization (WHO) and the United Nations Children’s Fund (UNICEF) in a set up designed to leverage each organization’s unique technical expertise in the global arena. Additionally, pharmaceutical companies that manufacture vaccines, donor and recipient governments, civil society organizations, research and technical institutes are also members of the alliance [14]. In this partnership model, Gavi does not place implementation support teams for its sponsored programs in countries but instead relies on both in- and out- of country partners under its partnership engagement framework (PEF) mechanism. WHO and UNICEF are the principal in-country implementation partners undertaking majority of PEF activities, while other organizations’ involvement depends on countries’ immunization programs’ capacity and needs [15].

Gavi financial support for new vaccine introductions (NVIs) began in 2000, with availability of funds for the combined diphtheria, pertussis (whooping cough), tetanus and Hepatitis B (DPT-HepB) vaccine. Subsequently the number of supported vaccines increased to the current 11; one of which is the Human Papillomavirus (HPV) vaccine [16]. Its inclusion in 2011, was however marked by an unprecedented requirement for countries to first implement a demonstration project prior to national scale-up, in order to gauge effectiveness of possible delivery models. The demonstration project was deemed necessary due to the novel target age group of 9–13 years that falls out of routine country national immunization programs’ (NIP) target age group of 9–24 months. Most LMICs do not have an adolescent specific health care service and therefore lack a health service delivery system for this target group [17]. HPV vaccine introductions were therefore being conducted in two phases, the demonstration project and the national roll out, each with its own set of decision making, planning and execution processes [18]. While Gavi has had a record of success in accelerating the national adoption of the other new vaccines [19], HPV vaccine introduction encountered a challenge of slow adoption. Twenty three countries completed demonstration projects by December 2016, but only 6 had advanced to national scale up phase by Dec 2017, a stark contrast to Gavi’s planned target of 8 nationwide HPV vaccine introductions by 2015 [20]. Despite Gavi’s effort to accelerate country uptake by removing the required demonstration project phase [21], only 18 LMICs countries have introduced HPV vaccines into their NIPs to date. The lagged adoption has been attributed to multiple factors, however an important barrier is the dearth in published country specific HPV vaccine demonstration project evaluation findings in the literature [22].

**Fig. 1:**
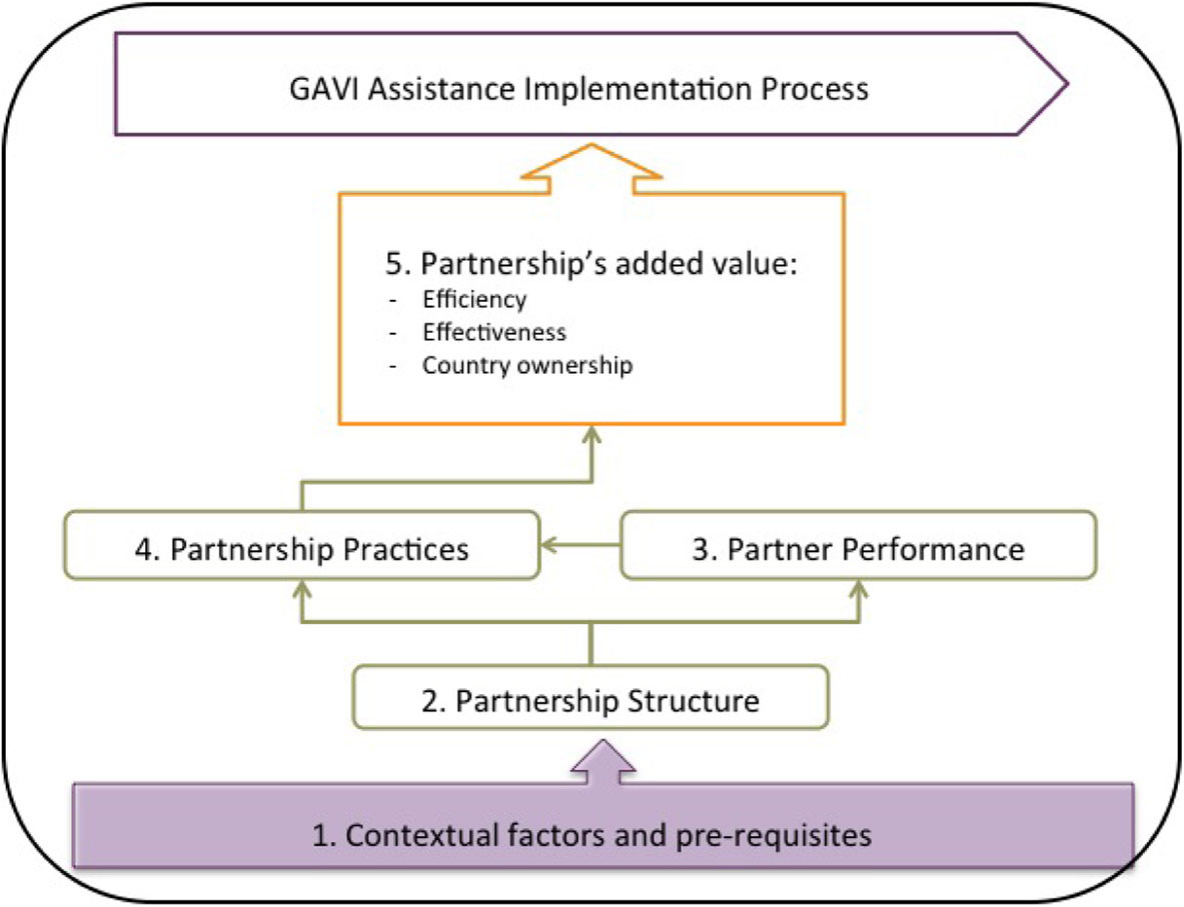
The Gavi FCE partnership evaluation framework

Social Network Analysis (SNA) is a modern sociology technique, that provides theory and tools for the visualization and measurement of interactive relationships amongst people or items. SNA is widely applicable to relational patterns created by any tangible or non-tangible content [23]. Because such patterns form network structures that may act as facilitators or constraints to the achievement of a networks’ objectives, SNA findings can be utilized to address a network’s structure bottlenecks or leverage its strengths [24]. Health services delivery researchers are increasingly harnessing SNA tools to understand diverse aspects of health systems. Organizational governance has proved to be an opportune topic for this type of inquiry which makes inconspicuous communication and collaboration patterns visible, thus enabling the study of key strategic and managerial decision-making information flows amongst important players within a health system [2, 25]. Despite this expansion in SNA utilization, its use in LMIC health systems governance research is still nascent [26, 27]. One aim of our study is to contribute to this expanding health systems SNA body of knowledge, through applying the techniques to study a complex vaccine delivery collaboration. We argue that SNA techniques can be used by global health implementation researchers to visualize a partnership network’s structure and measure its connectivity to understand how rapidly information diffuses through it. Furthermore we correlate SNA connectivity metrics scores with a network’s ability to effectively engage with and handle multiple scales (e.g. hierarchical levels and diverse sectors of the health system*),* combine and integrate different forms of knowledge as well as its capacity to anticipate and cope with uncertainties and surprises. We thus demonstrate how SNA techniques can be used for decision making when establishing a global health program implementation partnership. Our second objective is to fill the existing gap in documentation of country specific HPV demonstration project research findings in the literature. We present a case study conducted on Mozambique’s HPV vaccine delivery demonstration project. We leveraged the Gavi Full Country Evaluation (FCE) partnership framework (Fig. 1) [27] to test the contribution of network structure on the added value of the project’s partnership.

The remainder of this paper is organized as follows: Section 2 addresses the study setting, describes the Gavi FCE evaluation partnership framework dimensions and details the study design, data collection and data analysis procedures. In section 3 we describe the results in detail and then discuss them in section 4. Finally, we conclude the paper in section 5 and offer recommendations for national scaling up of Mozambique HPV vaccination.

## Methods

### Study setting

The 2014 to 2018 Gavi FCE, was a prospective in-depth assessment of inputs, outputs, outcomes and impact of its financial support to LIMC immunization programs. One key FCE research question explored the role of Gavi’s partnership model on decision making, planning, implementation and technical support in delivery of country immunization services. This paper presents findings from the case study undertaken in Mozambique in response to this partnership research question.

#### The case

The Mozambique HPV vaccine demonstration project, whose objective was to test a model for HPV vaccine delivery to girls aged between 9 and 13 years, was undertaken during 2 years, 2014 and 2015. It was implemented in three districts that were chosen to represent the three socioeconomically diverse geographical regions of the country. These were Manhiça, Manica and Mocímboa da Praia in Southern, Central and Northern regions respectively. Funding was obtained from Gavi for Manhiça district while the Government of Mozambique (GoM) funded the project in the other two districts. The tested delivery model was primarily school based whereby health workers administered the vaccine to girls in schools. Periods of 1 week were predetermined and utilized for the delivery of each dose. In the first year (2014) three doses were delivered in May, June and November in alignment with the then WHO recommended HPV vaccine administration schedule of 0, 1, and 6 months intervals. In the second year (2015) the WHO guidelines had changed to two doses to be given at 0 and 6 months and the vaccine was administered in June and November [28].

### The Gavi FCE partnership framework

Our study was oriented by the Gavi FCE partnership framework, which was developed through integration of concepts from public administration, organizational science and network analysis, resulting in five causally related partnership evaluation dimensions (Fig. 1). The framework differs from predecessor partnership evaluation frameworks, by placing the role of a partnership’s network structure at the core of its effectiveness. It postulates that effective partnerships require appropriate partnership network structures and proposes the empirical measurement of network structure, using SNA metrics, as a tool to inform partnership formation [27]. It hypothesizes that the framework’s structure dimension will be highly predictive of a partnership’s performance. This assumption is based on the social network diffusion theory [23] which explains how network structures act as either facilitators or barriers for adoption of novel practices. According to the theory, network-level structure outcomes for connectivity, efficiency and effectiveness, are determined by relationships between network actors. These relationships usually involve a relational content [23], such as information or resources, that can be measured using SNA metrics [23]. In this study technical assistance information, was the relational content measured, in order to determine the structure of collaboration relationships amongst Mozambique’s HPV vaccine demonstration project implementation partners.

### Study design

**Fig. 2:**
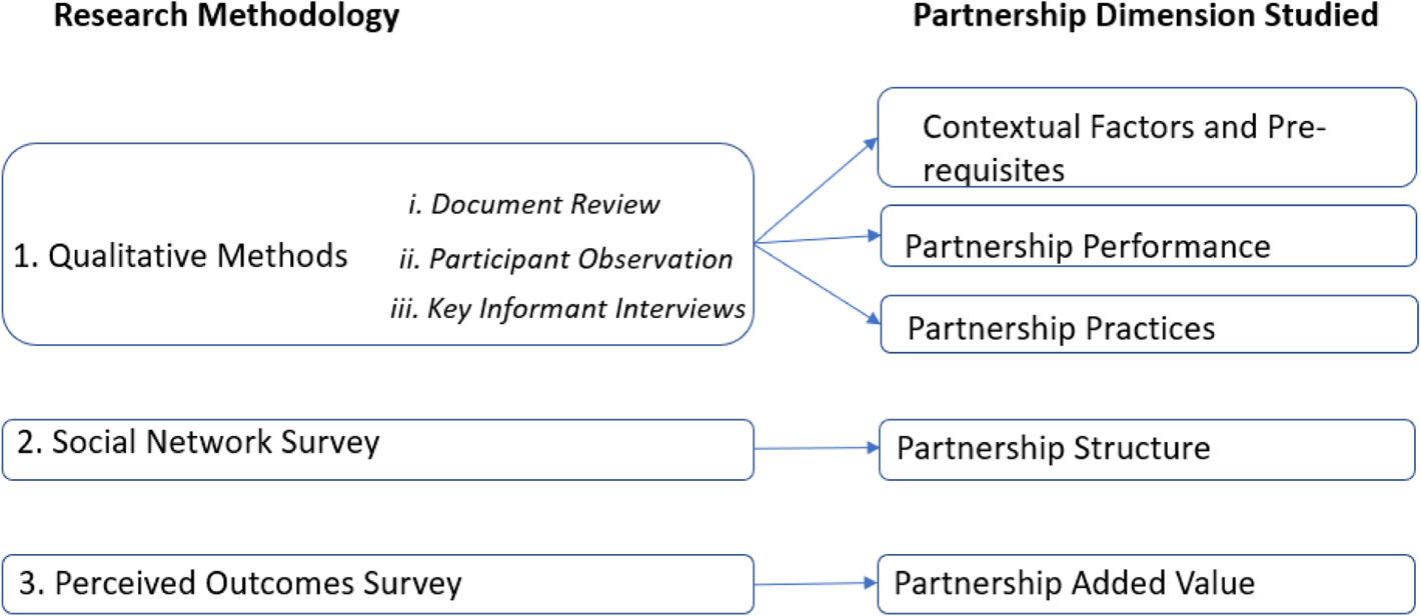
Research methods and partnership dimensions studied

Mixed methods were used to study the partnership evaluation framework’s five dimensions detailed in Table 1. Qualitative tools described the partnership’s contextual factors and prerequisites, partner performance and practices, so as to facilitate the understanding of its context, how and why partners perform as they do and how and why partnership practices are what they are. SNA measured the effectiveness and efficiency of the partnership’s network structure while a survey assessed partners’ perceptions about the added value of HPV vaccine implementation collaboration (Fig. 2).

**Table 1:** Gavi FCE partnership framework dimensions’ description

1. Contextual factors and prerequisites: Identification of the governmental and non-governmental institutions and organizations as well as individuals involved in implementation of the Mozambique HPV vaccine demonstration project. The origins of the partnership and how the broader political environment and the global Gavi Alliance partnership has influenced this. Other Mozambique contextual factors that have facilitated or blocked the successful formation and performance of this partnership. 2. Partnership structure: Understanding the composition of the partnership’s players and their relationships with each other, as well as the connections between the implementers of the HPV vaccine demonstration project in the country. 3. Partner performance: Exploration of how effectively partners meet their deliverables and the mechanisms for accountability. 4. Partnership practices: Investigation of the functionality of the whole partnership in terms of professional trust. Additionally, any formal and informal rules and processes that govern the partnership; partners’ competencies, capacities, roles and responsibilities, the partnership coordination mechanisms and management of the partnership. 5. Partnership’s added value: Determination of partners’ perceptions of outcomes of working together, namely efficiency, effectiveness and country ownership;

### Data collection

Institutional Review Board (IRB) approvals were obtained from University of Washington and Mozambique Ministry of Health prior to data collection. A team of Gavi FCE researchers, collected data at central level and in all three demonstration districts, during the 2 years that the HPV vaccine demonstration project was implemented.

#### Qualitative data

Three methods were used to gather qualitative data *i) document review ii) direct observations iii) key informant interviews (KIIs).* A broad range of documents were reviewed including all Mozambique HPV vaccine demonstration project vaccine grant proposals, HPV demo project budgeted plans, Mozambique Gavi business plan and partnership engagement framework, Gavi HPV vaccine demonstration project guiding documents, minutes of meetings held by the NIP and partners, notes from direct observations, terms of reference for the different HPV vaccine demonstration project related committees/technical working groups, NIP programmatic evaluation documents, NIP strategic plans, NIP audits and financial reports. Information gathered from document review were used to refine the questions in the KII topic guide.

Direct observations were conducted through participation [29] in all HPV vaccine demonstration project meetings ranging from NIP technical working group meetings; subcommittee meetings focusing on, for example, cold chain management; national immunization technical advisory group meetings (NITAG); and Inter Agency Coordination Committees (ICC) meetings. Additionally, events such as trainings, supervision visits, the official launch and all implementation activities for HPV vaccine demonstration project in the three demonstration districts were attended. Information gathered from direct observations that respond to the evaluation aspects of the five dimensions of the partnership framework were used to further refine the questions in the KII topic guide.

KII respondents were identified through a two-pronged sampling approach. First, was the comprehensive approach targeting all known NIP stakeholders and second the chain referral (snow ball) approach targeting other key informants (KIs) as they were referred by those already sampled [30]. Known stakeholders refer to institutions and organizations that had been distinguished during document review and direct observations. Samples were drawn from the following groups within these identified entities: health facility immunization staff, district immunization staff, district education staff, provincial immunization staff, provincial medical heads, central MOH level immunization program staff, staff from research institutions, non-Governmental organizations (NGOs), bilateral and multilateral institutions that supported the NIP during all the HPV vaccine demonstration project phases. A semi-structured open-ended interview guide was developed and used for all respondents with probing techniques being applied whenever the need arose. Interviews were conducted in the place of convenience to the key informant, usually in their office. Appointments were arranged through both email and phone. Consent forms and topic guides were shared with respondents prior to interviews which were documented through note taking and audio recording.

#### Social network and perceived outcomes data

Both SNA and perceived outcomes data were collected through a single two-section structured questionnaire (Additional file 1). The positional strategy [23] was used to specify the network boundary with actor identification being based on their positions in institutions and organizations that were involved in HPV vaccine demonstration project implementation. Each actor was asked: “With whom did you work with on the HPV vaccine demonstration project?” This process is referred to as a ‘name generator’ in social network analysis [23]. For documentation of the relational content, survey respondents were asked whether they had received or provided technical assistance. In order to measure tie strength respondents were asked to rate their level of professional trust and satisfaction on a scale of one to five. The professional trust question was “when working with the individual you have named, do you trust that he/she has the ability to respond to what you requested for and to do and complete what they have promised to do?”

The perceived outcomes section contained a set of questions about the KIs’ perceptions of their working relationships with other HPV vaccination implementing partners. These questions were adapted from Provan et al. [31] and organized according to perceived benefits and drawbacks of working together. Respondents were asked to choose if the benefit or drawback had occurred or had not occurred when they worked together as a partnership with HPV vaccine demonstration project implementation partners. Probing was employed to capture reasons for the choice provided by the informant and notes taken to document responses.

### Data analysis

The units of analysis for the qualitative segment of our study, were the individuals, institutions and organizations directly involved in the implementation of the Mozambique HPV vaccine demonstration project. Audio recorded data was transcribed into texts by known professional transcribers with experience in transcribing other audio recorded data for the Mozambique Gavi FCE. Nvivo software was used to facilitate a theme-based analysis as per the five dimensions of the Gavi FCE partnership conceptual framework. A preliminary codebook was developed and responses to the partnership framework dimension’s research questions sought and given their own code. Following coding, a report of each code was produced, read, and re-interpreted resulting in a synthesis of findings related to each dimension.

Two sets of hypotheses, listed in Table 2, guided SNA measurement of the partnership network structure. The first set relate network structure connectivity to WHO health system governance characteristics, an approach originally described by Blanchet et al. [32]. The second set stem from SNA theory of diffusion and relate network structure to network efficiency and effectiveness [21].

**Table 2:** Social network analyses hypotheses and metrics

**WHO health system governance characteristics hypotheses and measures for network connectivity** *Characteristic 1: Capacity to effectively engage with and handle multiple scales (*e.g. *levels and sectors of the health system)* Reachability: Networks with a high level of reachability have the ability to access various sources of information. Distance: The shorter the distance between the actors the faster the diffusion of information. Networks with short average path length are more likely to facilitate the widespread diffusion of information *Characteristic 2: Capacity to anticipate and cope with uncertainties and surprises* Centralization: A centralized structure has a higher capacity to coordinate actors and provide rapid response Betweenness: Rapid response occurs when the key actors have the ability to reach all the players in the network *Characteristic 3: Capacity to combine and integrate different forms of knowledge* Reachability: The diversity of technical knowledge can be achieved through relationships with actors that belong to other spheres or subnetworks. Density: Dense networks are more likely to facilitate the transfer of information however actors in a dense network have difficulty in accessing diverse forms of knowledge **Theory of network diffusion hypotheses and measures for network efficiency and effectiveness** Structural holes: Network with more structural holes is more efficient for the diffusion of information Redundancy: Less redundancy means a more efficient network for the relaying of information Homophily: Novel information is more likely to enter heterogeneous networks while homophily in a network can be a barrier to accessing new information	

The individual was the unit of analysis for both social network and perceived outcome analyses. Data collected from the network survey was entered into a spreadsheet matrix in MS Excel. Each reported working relationship, or “tie,” was weighted according to the key informant’s rating of relationship trust while the technical assistance exchange tie was entered as binary. The resulting spreadsheet contained three asymmetrical, undirected network matrices which were imported into UCINet software [33] for analysis. Descriptive network maps were created using the NetDraw [34] application within UCINet software with nodes being color coded according to node attributes from the survey. Tests were run for each SNA metric associated with the hypotheses behind our SNA (Table 2). Five metrics namely *reachability, distance, centralization, betweenness* and *density* measured network structure’s connectivity while three metrics, s*tructural holes, redundancy* and *homophily* measured its efficiency and effectiveness. SNA metrics that measure the networks structure efficiency and effectiveness are also referred to as network outcome measures.

Data from the perceived benefits and drawbacks survey section was entered in the same excel spreadsheet as the network data and proportional scores calculated for each survey response. In addition, the benefits and drawbacks were stratified into effectiveness, efficiency and country ownership and a mean score calculated for each stratum.

We utilized an iterative triangulation process, in which all the three types of data were analyzed and reanalyzed, to construct patterns and facilitate interpretation.

## Results

### Descriptive statistics

A total of 40 KIIs were conducted at national, provincial, district and health facility levels in the three HPV vaccine demonstration district sites in the country (Table 3). Of these study participants, 34 responded to the social network and perceived outcomes surveys. Majority of those interviewed were from the Ministry of Health (MOH) (55%) and the Ministry of Education (MOE) (15%) with a larger proportion (35%) being from the district level.

**Table 3:** Organization affiliation of study participants

Organization Type	Number	%
MOH NIP	22	55
*National*	*5*	*12.5*
*Provincial*	*6*	*15*
*District*	*11*	*27.5*
MOE	6	15
*National*	*1*	2.5
*Provincial*	*2*	5
*District*	*3*	7.5
Multilateral	3	7.5
CSO/NGO	4	10
Research Institute	3	7.5
Bilateral	1	2.5
Pharmaceutical	1	2.5
**Total**	40	100%

### Gavi FCE partnership framework dimensions’ detailed results

#### Contextual factors and prerequisites

We found that, Gavi plays a key role in driving Mozambique’s HPV vaccination delivery partnership, nevertheless other significant contextual factors also emerged as key drivers of the partnership. These are; the country’s first lady’s involvement as a champion, Gavi’s requirement for the NIP to demonstrate feasibility of its HPV vaccine delivery model and schools as the primary location of HPV vaccine administration.

Gavi’s partnership model, is the main driver of the country’s national immunization collaboration which according to interviewed informants historically stemmed from the country becoming a beneficiary of Gavi grants in 2001. The two core Gavi partners WHO and UNICEF, whose task within the Alliance, are to provide in-country implementation support, were referred to by NIP staff as the “traditional partners”. Each organization is known for specific roles, WHO for technical guidance and UNICEF for logistics and supplies, a fact that we learned has influenced the NIP to adopt the practice of allocating specific roles to potential partner organizations that express interest to collaborate with it.

*“We are used to working with WHO for technical guidance. UNICEF they usually support us for vaccine logistics and supply” NIP.*

Two other organizations, VillageReach an international non-governmental organization (NGO) and Fundação para o Desenvolvimento da Comunidade (FDC) a local organization were also considered as “usual” NIP partners and had supported immunization activities for more than 5 years prior to the HPV vaccine demonstration project launch. Further investigation revealed that that these two organizations had strong ties to Gavi. VillageReach is an American based organization whose funding comes from the BMGF, a founding partner of Gavi. In addition, one of its founding board members has been Gavi’s chief executive officer for 7 years, from 2011 to date. FDC’s founding president had also previously been the chair of Gavi’s board for a decade, from its inception in 2001 until 2009. Results qualitative data triangulation, revealed the importance of these Gavi links, to VillageReach’s and FDC’s participation as NIP’s partners in Mozambique’s HPV vaccine delivery demonstration project implementation.

*“Invitation for VillageReach (to be NIP partner) was explicit. Especially after the head of NIP understood VillageReach’s work and its relationship with Gavi. She (NIP head) was initially new. The chief executive officer of Gavi used to be board member of VillageReach from the inception of VillageReach. We were invited when they realized the relationship we had with Gavi and the information that VillageReach possesses about Gavi”* (Civil Society Organization).

“*… .for example FDC was already interested in HPV vaccination introduction, from before because its president Machel was a member of the Gavi board for many years”* (Research Institute).

In the year prior to the launch of the HPV vaccine demonstration project, Mozambique’s then first lady assumed leadership of the Forum of African First Ladies Against Breast & Cervical Cancer and hosted a conference in Maputo, the country’s capital [35]. This position propelled her to become a HPV vaccination champion in both the continent and in-country, with subsequent impact on HPV vaccination pilot partnership. Her championship position influenced the NIP and MOH to take on a leadership role as opposed to the participatory one that both entities had assumed during HPV vaccine grant application writing. Furthermore, MOH leveraged government funds to carry out demonstration activities in two additional districts, instead of only one district that Gavi was funding. Another outcome of MOH leadership, was the disbursement of HPV vaccine introduction grant (VIG) funds from Gavi through MOH and not to Gavi in-country partners, WHO and UNICEF as had happened previously, with pneumococcal vaccine (PCV) introduction grant. KIs noted that, by assuming a leadership role, the MOH became better placed to negotiate how Gavi funds would be received in-country. The expansion in number of demonstration project sites impacted on the HPV vaccination pilot project partnership because it led to increase number of players.

A couple of other contextual factors unique to HPV vaccine delivery model, led to a partnership which differed compositionally from the one observed during other Gavi funded new vaccine introductions in Mozambique. First was Gavi’s requirement for the inclusion of an assessment component to examine feasibility of the delivery model that would be tested during the demonstration project. For this purpose, two research institutions, the Manhiça Research Center (CISM) and the country’s National Institute of Health (INS) were included in the partnership, to lead assessments in Gavi and Government funded districts respectively. The second factor was the nature of delivering HPV vaccine in schools necessitating the involvement of MOE in the partnership. MOE personnel had to carry out specific HPV vaccination activities at all levels from national to provinces, districts and schools.

#### Partnership structure

The SNA results for *reachability, distance, centralization, betweenness, density, structural holes, redundancy, homophily and average trust* are shown in Table 4. The network structure was found to contain a total of 50 actors (nodes) and 164 ties.

**Table 4:** Network topology measures

Metric	Value	Hypothesis (from Table 2)
Nodes	50	
Ties	164	
Distance (average path length)	2.5	Networks with short average path length are more likely to facilitate the widespread diffusion of information
Reachability	1	Networks with a high level of reachability have the ability to access various sources of information
Centralization	0.483	A centralized structure has a higher capacity to coordinate actors and provide rapid response
Betweenness	37.24	Rapid response occurs when the key actors have the ability to reach all the players in the network
Density	0.1338	Dense networks are more likely to facilitate the transfer of information however actors in a dense network have difficulty in accessing diverse forms of knowledge
Structural holes	85%	Network with more structural holes is more efficient for the diffusion of information
Redundancy	70%	Less redundancy means a more efficient network for the relaying of information
Homophily E-I index	0.195	Novel information is more likely to enter heterogeneous networks while homophily in a network can be a barrier to accessing new information
Average tie weight (reported trust)	4.056	

**Fig. 3:**
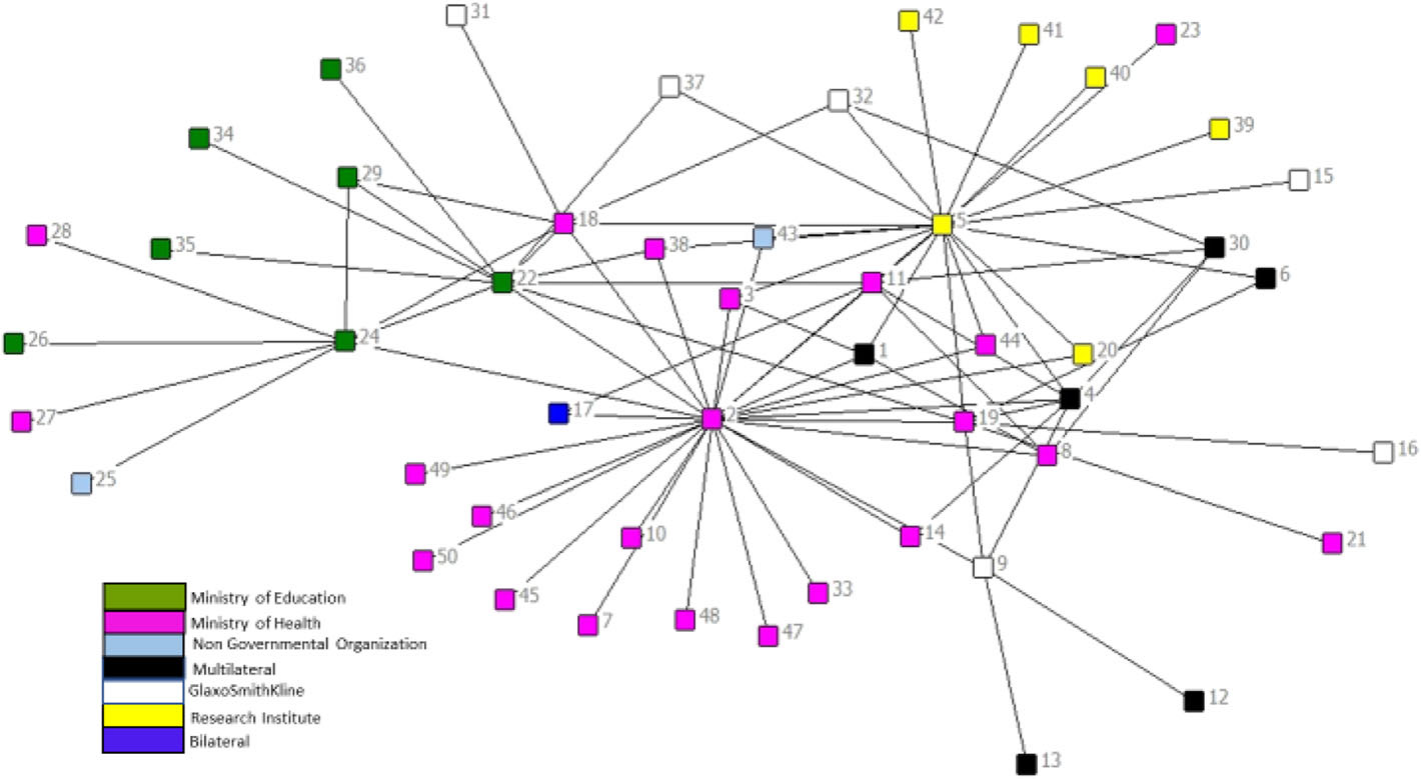
Mozambique HPV vaccine demonstration project partnership network structure

The *reachability* score is 100% meaning that there is at least one path connecting all actors in the network and each can be reached from whichever point one starts from (Fig. 3). The *distance* score, which is defined as the average number of edges in the shortest path between pairs, at 2.52 is short. Shorter distance in SNA is commensurate with faster and more accurate information flow. Combining the two SNA metrics scores with our first WHO health system governance characteristic hypotheses (Table 2) we can infer a partnership that has the capacity to effectively engage with and handle multiple scales. The perceived outcome survey scores corroborate this finding (Table 5). At 97.6%, the effectiveness average score was the highest of all outcome survey mean scores. Furthermore, we noted 100% partners’ agreement to three perceived outcome questions; 1) HPV vaccination partnerships ability for better execution, 2) better quality and improved response to challenges when they worked with multiple types of entities and 3) organizational hierarchical levels.

**Table 5:** Perceived benefits of partnership (*n* = 34)

Benefits:	% of respondents who agreed
**Effectiveness**	
Better able to execute activities	100%
Planned activities were executed with greater quality	100%
Better able to identify the need for, and to acquire additional support	97%
Better able to respond to existing challenges, or those that arose during the process	100%
Increased sustainability of immunization program	91%
Mean Effectiveness	97.6%
**Efficiency**	
More timely execution of planned activities	94%
Leveraged each organizations’ comparative advantages	88%
Reduction in financial cost of process	74%
Better allocation of each organization’s financial resources	62%
Mean Efficiency	79.5%
**Country Ownership**	
Increased country ownership	79%
Increased transparency among partners	91%
Increased accountability among partners	74%
Increased legitimacy of decisions made	94%
Increased fairness of decisions made	91%
Mean country ownership	85.8%
**Drawbacks:**	
**Effectiveness**	
Strained relations within my organization	9%
Created competition and conflict among member organizations	6%
Mean Effectiveness drawbacks	7.5%
**Efficiency**	
Unnecessary management burden on my organization	24%
Loss of control/autonomy over decisions	6%
Forced us to make decisions in a way which was not natural/typical for our organization	21%
Mean Efficiency drawbacks	17%
**Country Ownership**	
Not enough credit given to my organization	24%
Total Country Ownership drawbacks	24%

For the second WHO health system governance characteristic hypotheses, the *centralization* score was found to be neither low nor high at 48%, and average betweenness was 37.24 with a large standard deviation of 110.1. These scores are consistent with the network’s outdegree statistics that revealed three outlier actors numbers 2, 5 and 24 (Fig. 3), around which the network is centralized. While actor number 2 had the highest outdegree score, this *centralization* value, means that the partnership is not highly centralized around 1 focal actor (e.g. EPI program). In addition, the network structure connectivity scores support the existence of effective relationships between these three key actors, indicating that the partnership could coordinate and respond rapidly to challenges. When triangulated with perceived outcomes survey results, we found a concordance as 100% of respondents, answered affirmatively for the question on their perception of HPV vaccination partnership’s capacity to respond to challenges which had arisen during project implementation processes (Table 5). Qualitative data further supported the finding (see quotation below).

*“The involvement of many organizations was very advantageous because we as the district directorate of health would not have been able to undertake all the activities within the short time that we had to prepare. The partners and their support helped us to reach where we would not have reached, for example sometimes they gave us fuel when we didn’t have and even one hired a boat to reach some islands” (District Health Directorate).*

The third WHO health system governance characteristic is the capacity to combine and integrate different forms of knowledge whose hypotheses (Table 2) are based on *density* and *reachability* across different spheres and entities. According to SNA diffusion theory, actors in very dense networks have difficulty in accessing diverse forms of knowledge; however density is important for the effective transfer of complex knowledge [36]. This network has a low *density* score of 0.1338, which taken together with the observed diversity of types of entities in the network, distinguished by different colors in Fig. 3, and the 100% *reachability* score, mean that actors in this partnership are well positioned to receive new types of information. Further support for this finding comes from contextual qualitative data. KII respondents talked of the implementing partnership for HPV vaccine, being compositionally different from implementing partnerships for traditional NIP vaccines which target children below 22 months of age compared to HPV vaccine’s novel target age group of 9–13 years.

*“The target group for HPV vaccine is different so we have to work with different collaborators, for example the ministry of education and partners in the community that helped us to pass the message” (District Health Directorate).*

#### Partnership performance, practices and outcomes

The last three dimensions are interpreted here jointly because our conceptual framework postulates that a partnership’s structure determines its performance, practices and consequently the outcomes. SNA measures have so far revealed a network characterized by high overall connectivity that is favorable for rapid and widespread diffusion of information. In addition, trust within the partnership is very high as evidenced by the high tie strength average score of 4.056 which is very close to the upper limit of five on the scale that respondents had been asked to rate professional trust on. Network outcome SNA measures (described in Table 2) unveiled an efficient network structure characterized by a high number of *structural holes* and less *redundancy*. Majority (85%) of the nodes in the network require only 2 or 3 paths to reach them and 70% have only one way to connect with other nodes meaning that *redundancy* is only 30%. The network was also found to be heterogenou*s* with a *homophily* E-I index scores of 0.195.

On triangulation with qualitative and perceived outcomes data so as to facilitate interpretation we found that these topological features of the network influenced partnership practices and subsequently the outcomes. The partnership’s structure heterogeneity significantly influenced partnership practices. The avoidance of duplication of activities or partner organizations focusing on the same activities in the same geographical area emerged as very key to the effectiveness and efficiency of this partnership. Respondents at all levels emphasized the role of regular meetings in the form of a formalized NIP technical working group (TWG) at national level and regular meetings chaired by the head of health at provincial and district levels. Specific roles for specific partner organization was another attribute that respondents repeatedly stated. Remarkably each partner organization was known for a specific role in this partnership. Beyond WHO and UNICEF’s earlier stated roles VillageReach was known as a logistical support partner, FDC for community mobilization and GlaxoSmithKline (GSK) usually supported printing of training material and health worker job aids. Perceived outcomes survey scores added strength to these findings with 88% of respondents agreeing that the partnership leveraged each partner organizations’ comparative advantages.

“*… … .the duplication of activities does not occur because each organization presents its activities to the directorate and the directorate tells them where they can work. Apart from that we have regular coordination meetings”* MOH provincial Directorate.

Lower agreement perceived outcome scores were noted for the questions on country ownership (79%), reduction in financial cost of process (74%) and better allocation of each organization’s financial resources (62%). Many district level respondents expressed their dissatisfaction in the sufficiency of funding that had been availed to them for HPV vaccination activities, with the situation being worse in the districts that were not funded by Gavi and had to depend on only MOH funds. While some respondents noted that partner organizations had stepped in and helped a lot, especially with in kind donations, many talked of lack of funding for outreach activities for finding non-school attending girls in communities as a major challenge. Correspondingly, the highest perceived outcomes drawbacks score was that of country ownership at 24%. Several issues were mentioned regarding country ownership but featuring prominently was the preference for a country based partner organization. Respondents expressed their unhappiness on the inclusion of a non country-based partner in the HPV implementation partnership. Language barrier and lack of contextual knowledge were mentioned as some of the problems of the specific technical assistance that was provided by the particular partner who was considered foreign. The lack of participation in regular technical working group meetings was also noted as a hindrance to individuals based outside the country contributing effectively to the partnership. This is because TWG meetings was the forum where most of the partnership communication took place with updates on progress on processes being made, important discussions taking place and key decisions being made during the meetings. Short visits to the country to provide technical assistance were said to be ineffective by survey respondents and were even blamed for having largely contributed to the delay in the accomplishment of some HPV vaccination pilot implementation phase deliverables.

## Discussion

Delving into the Mozambique HPV vaccination implementation stakeholder relationships in this case study, has revealed the importance of balance between donor and government influence, the necessity for adaptability to changing needs and the value of country ownership for an effective vaccine delivery partnership.

We observed a partnership that was equally driven by the MOH as well as the influence of the global Gavi partnership model. The balance between Gavi and Government of Mozambique influences during the implementation of this pilot led to remarkable effectiveness and efficiency results. The perceived outcome survey mean effectiveness score was quite high at 97.6% and the mean efficiency was 79.5%. Partnership composition and practices were equally driven by both entities resulting in a complementary relationship that engendered a favorable collaborative environment in which the best that each institution had to offer was leveraged. The Gavi partnership model of having specific core partner roles guided the HPV partnership to adapt these principals and as a result each organization’s competencies were optimized and redundancies in terms duplication of partner activities were avoided. These conditions created a conducive environment for the successful testing of a challenging vaccine delivery model for a novel target group in three different Mozambican socioeconomic contexts with the outcome of important knowledge being gained for national scale up planning. Had Gavi’s influence been dominant and the original Gavi decision followed, the HPV vaccination pilot would have taken place in only one district representing only one Mozambican economic and religious context and learnings from the different contexts that the other two districts offered, would have been missed. Similar significance of country level influence in decision making for introduction of new vaccines is documented in the literature [37, 38].

Another salient finding emerging from this study is the importance of a flexible partnership to adequately respond to unpredictable events and evolving priorities. The expansion of the Mozambique demonstration project that was originally planned for just one district to three districts happened in a short period of time, within just 6 months prior to the launch of the demonstration project. The ability for the partnership to adapt and bring on and integrate new members to the implementation collaboration was outstanding. This finding not only agreed with the SNA hypotheses that we had adapted from Blanchet et al. regarding the second characteristic of the WHO health system governance characteristic number two (Table 2) but with our SNA results too. Our results, which align with previous study findings [39, 40], clearly demonstrate that a partnership whose structure is moderately centralized, around few key diverse players, has a higher capacity to coordinate actors and provide rapid response. Similarly, rapid response occurs when the key diverse players have the ability to reach all actors across the different entities in the network. In addition, another learning was the function of an innovative intervention requiring additional roles and further leading to the expansion of the partnership. Again, the partnership was malleable and successfully integrated the necessary research institutions and ministry of education for the purpose.

The third and final outcome measure of our study was the country ownership for which perceived outcome survey score at 79.5% at was among the lower scores. A salient factor that was elucidated was how the location, in or out of country, of a technical assistance provider affects the outcome of their support. Specific barriers that were encountered by the out of country international partner and which we have highlighted in our results section, are particularly informative and relevant for Gavi to consider when assessing potential technical assistance providers for countries in the current era of the partnership engagement framework (PEF).

In applying the Gavi FCE partnership framework we have found it to be a particularly useful tool for conducting a comprehensive partnership evaluation. This is because unlike previous predecessor partnership evaluation frameworks, namely the traditional and Brinkerhoff [41], the FCE framework approach is more wholistic resulting in consideration of an expanded set of partnership characteristics which influence partnership performance outcomes. While all three frameworks invariably describe the causal chain nature of the partnership characteristic from the contextual factors and prerequisites to the outcomes, the inclusion of the partnership structure together with the provision for the use of SNA tools to study it, highly enhanced the framework. These components of the FCE framework offer the possibility of unmasking partnership attributes that may not be overt.

The utilization of SNA tools in our study enabled us to visualize the otherwise invisible constellation of relationships that make up Mozambique’s HPV vaccine delivery partnership network. The image of the network structure facilitated a rapid identification of key players within the implementation collaboration. As in other studies [42, 43], SNA measurements demonstrated the partnership network structure’s effectiveness and efficiency in information flow and innovation adoption. Such information can only be attained using SNA methods thus making it a powerful methodology to inform other vaccine delivery endeavors and health program partnerships. Furthermore, the FCE framework guided our utilization of mixed methods which allowed for a broader analysis and exploration of the partnership. We therefore recommend the use of the Gavi FCE framework for evaluation of global health partnerships.

### Strengths and weaknesses

Our study’s participation rate was quite high with 40 individuals from different entities and different health system levels responding to both the network and perceived outcomes survey. This is a relatively large sample size especially for the qualitative data component; however, the accuracy of network data rely on a full census of a network and this study’s response rate may lead to underestimations of network density in particular. Ties beyond the set boundary were not explored and there might be missing data that was not captured. Mixed methods captured different types of data that were triangulated, and this facilitated interpretation.

## Conclusion

Global health implementation draws together diverse international entities for the purpose of enhancing health intervention adoption and institutionalization. The resulting complex collaborations’ efficiency and effectiveness can be evaluated so as to provide knowledge for decision makers tasked with creating such partnerships. Harnessing mixed research methods with the use of several types of qualitative methods including document review, participant observation and key informant interviews in combination with SNA and perception surveys can highly augment the assessments. This study’s scientific contribution is the illustration of how disparate research methods can be utilized together to conduct in-depth examination of pertinent partnership dimension components. Even though the results of the study may have limited applicability in settings whose vaccination services are organized differently from those of Mozambique, its finding are generalizable to low and middle-income countries with similar socioeconomic features.

Finally, the study revealed that while there is room for improvement on the country ownership aspects of the partnership, the expanded, diverse and inclusive collaboration of institutions and organizations that implemented Mozambique’s HPV vaccine demonstration project was effective and efficient and we recommend that the country adapt a similar model during national scale up. Our results also can also be used to orient Gavi’s PEF partner selection for Mozambique and other countries in a similar context. For future work, the methods utilized in this study will be replicated by our implementation research teams working in other health program areas in Mozambique and other LMICs.

## Supplementary information

**Additional file 1.**

## Data Availability

The datasets generated and analyzed during this study are not available because the interview and survey files are personally identifiable. The social network and outcome survey data files contain names and key informant interview files would be identifiable to a person familiar with the Mozambique health system given that study participants were identified by their specific roles and responsibilities within the health system.

## Abbreviations

BMGF: Bill and Melinda Gates Foundation
CISM: Centro de Investigação de Saude da Manhiça
CSO: Civil Society Organization
FCE: Full Country Evaluation
FDC: Fundação para o Desenvolvimento da Comunidade
GHPs: Global Health Partnerships
GOM: Government of Mozambique
GSK: GlaxoSmithKline
HPV: Human papillomavirus
ICC: Inter-agency Coordination Committee
INS: Instituto Nacional de Saúde
KIIs: Key Informant Interviews
LICs: Low Income Countries
LMICs: Low and Middle-Income Countries
MOE: Ministry of Education
MOH: Ministry of Health
NGOs: Non-Governmental Organizations
NIP: National Immunization Program
NVIs: New Vaccine Introductions
PCV: Pneumococcal Vaccine
PEF: Performance Engagement Framework
SNA: Social Network Analysis
TWG: Technical Working Group
UNICEF: United Nations Children’s Fund
VIG: Vaccine Introduction Grant
WHO: World Health Organization
