# Supplementary material for: Global health systems partnerships: a mixed methods analysis of Mozambique’s HPV vaccine delivery network actors
Source: BMC Public Health. 2020 Jun 5;20:862. doi: 10.1186/s12889-020-08958-1 (PMC7275554; doi:10.1186/s12889-020-08958-1)
Supplement: Supplementary file 1 — Additional file 1. [file 12889_2020_8958_MOESM1_ESM.docx]

**Mozambique Gavi FCE SNA and Perceived outcome survey questionnaires**

Respondent’s name: _________________________________Respondent’s organization: _________________________

Respondent’s job title: _____________________________________Location (city): _________________________________

Is respondent’s organization an international, national, or sub-national organization? : ______________

Number of years respondent has been at his/her present organization: ­­­­­­­­­­­­___________

Interview Date: ________________Name of Interviewer: _______________________________________________________

**Introduction**

My name is …….................................................... I am a member of the Gavi evaluation team that is evaluating the processes, networks and systems for the delivery of vaccines in country.

The topic we would like to address in today’s interview is:

Mapping of the networks of those involved in the implementation of Gavi funded streams (Rotavirus vaccine (RV), IPV, MSD, HPV and HSS) in the last 12 months.

1. Can you tell me the names of the people that you worked together with in the implementation of Gavi funded streams in the last 12 months. The streams we are referring to are RV, IPV, MSD HPV and HSS. List the name of the individual, organization or government ministry (MOH, MOF etc) and the department and title of the person as well as the Gavi stream you worked together on*.*

*[Allow respondent to list all names, giving them time and silence to think of additional names. By the end of the listing if Ministry of Education and provinces have not been mentioned, probe about these where appropriate].*

1. I would like you to reflect on the overall “professional trust” you have for each listed individual and give scores in the sheet below. When we say ‘professional trust,’ we mean your confidence that he/she can respond to what you have requested, that they will complete what they have agreed to do and that they will perform it well. Mark with a circle the appropriate number on the provided survey form.

1 = no confidence; 2 = some confidence; 3= satisfactory confidence; 4 = high confidence; 5 = very high confidence

1. Now I would like to know if you provided or received technical assistance (TA) from the individuals you have listed. By technical assistance we mean the transfer of knowledge or competencies. Circle a “P” for “I provided” or an “R” for “I received”.
2. Next we would like to know your level of satisfaction with the provided or received technical assistance. Mark with a circle the appropriate number in the provided survey form.

1 = High dissatisfaction; 2 = dissatisfaction; 3 = fair satisfaction; 4= high satisfaction; 5 = very high satisfaction.

*When the respondent completes filling in the form, probe around negative TA satisfaction and professional trust scores. Ask them to give reasons for negative scores. Find out how the given negative example has impacted on the partnership*

| **Individuals the respondent worked with** | **New vaccines** | **HPV** | **HSS** | **Professional trust** | **I provided/requested technical assistance to/from them** | **Satisfaction with TA provided/received** |
| --- | --- | --- | --- | --- | --- | --- |
| (Name, organization, department, title) | (Mark with an X) | | | (Circle) | (P/R) | (Circle) |
| Name:  Org:  Department: |  |  |  | 1 2 3 4 5 |  | 1 2 3 4 5 |
| Name:  Org:  Department: |  |  |  | 1 2 3 4 5 |  | 1 2 3 4 5 |
| Name:  Org:  Department: |  |  |  | 1 2 3 4 5 |  | 1 2 3 4 5 |
| Name:  Org:  Department: |  |  |  | 1 2 3 4 5 |  | 1 2 3 4 5 |
| Name:  Org:  Department: |  |  |  | 1 2 3 4 5 |  | 1 2 3 4 5 |
| Nome:  Org:  Departamento: |  |  |  | 1 2 3 4 5 |  | 1 2 3 4 5 |
| Name:  Org:  Department: |  |  |  | 1 2 3 4 5 |  | 1 2 3 4 5 |
| Name:  Org:  Department: |  |  |  | 1 2 3 4 5 |  | 1 2 3 4 5 |
| Name:  Org:  Department: |  |  |  | 1 2 3 4 5 |  | 1 2 3 4 5 |
| Name:  Org:  Department: |  |  |  | 1 2 3 4 5 |  | 1 2 3 4 5 |
| Name:  Org:  Department: |  |  |  | 1 2 3 4 5 |  | 1 2 3 4 5 |
| Name:  Org:  Department: |  |  |  | 1 2 3 4 5 |  | 1 2 3 4 5 |
| Name:  Org:  Department: |  |  |  | 1 2 3 4 5 |  | 1 2 3 4 5 |

I would like to know the benefits and drawbacks of working together with other individuals, organizations, or departments in a group / (NIP partnership). Mark in the box below whether you think that the listed benefit or drawback occurred. Check only one box for each benefit or drawback

| **Benefits:** | **Occurred** | **Did not occur** |
| --- | --- | --- |
| 1. Better able to execute activities | ☐ | ☐ |
| 1. More timely execution of planned activities | ☐ | ☐ |
| 1. Planned activities were executed with greater quality | ☐ | ☐ |
| 1. Better able to identify the need for, and to acquire additional support | ☐ | ☐ |
| 1. Better able to respond to existing challenges, or those that arose during the process | ☐ | ☐ |
| 1. Better allocation of each organization’s financial resources | ☐ | ☐ |
| 1. Reduction in financial cost of process | ☐ | ☐ |
| 1. Leveraged each organizations’ comparative advantages | ☐ | ☐ |
| 1. Increased sustainability of immunization program | ☐ | ☐ |
| 1. Increased country ownership | ☐ | ☐ |
| 1. Increased transparency among partners | ☐ | ☐ |
| 1. Increased accountability among partners | ☐ | ☐ |
| 1. Increased legitimacy of decisions made | ☐ | ☐ |
| 1. Increased fairness of decisions made | ☐ | ☐ |
| 1. Other benefits: |  |  |
|  |  |  |
| **Drawback:** | **Occurred** | **Did not occur** |
| 1. Unnecessary management burden on my organization | ☐ | ☐ |
| 1. Created competition and conflict among member organizations | ☐ | ☐ |
| 1. Loss of control/autonomy over decisions | ☐ | ☐ |
| 1. Strained relations within my organization | ☐ | ☐ |
| 1. Not enough credit given to my organization | ☐ | ☐ |
| 1. Forced us to make decisions in a way which was not natural/typical for our organization | ☐ | ☐ |
| 1. Other drawbacks: | ☐ | ☐ |
